# Secondary Vitrectomy with Internal Limiting Membrane Plug due to Persistent Full-Thickness Macular Hole OCT-Angiography and Microperimetry Features: Case Series

**DOI:** 10.1155/2020/2650873

**Published:** 2020-09-21

**Authors:** Dominika Wrzesińska, Katarzyna Nowomiejska, Dominika Nowakowska, Mario Damiano Toro, Vincenza Bonfiglio, Michele Reibaldi, Teresio Avitabile, Robert Rejdak

**Affiliations:** ^1^Department of General Ophthalmology, Medical University of Lublin, Lublin, Poland; ^2^Faculty of Medicine, Collegium Medicum Cardinal Stefan Wyszyński University, Warsaw, Poland; ^3^Department of Experimental Biomedicine and Clinical Neuroscience, Ophthalmology Section, University of Palermo, Palermo, Italy; ^4^Department of Ophthalmology, University of Catania, Catania, Italy; ^5^Department of Surgical Sciences, Eye Clinic Section, University of Turin, Turin, Italy

## Abstract

**Purpose:**

To study the features in OCT-angiography and microperimetry in eyes with persistent full-thickness macular hole (FTMH) closed with the secondary plana vitrectomy (PPV) with autologous internal limiting membrane (ILM) plug.

**Methods:**

Secondary PPV was performed with closing the persistent FTMH with ILM plug, C_3_F_8_ tamponade, and face-down positioning. Four patients were followed for 6 months with best corrected visual acuity (BCVA) measurement, SD-OCT and OCT-A, and microperimetry. The results were compared with the fellow eye; in two patients, it was the healthy eye, and in two remaining eyes, successfully closed FTMH after primary PPV.

**Results:**

ILM flap was integrated in all cases with *V*-shape of closure, and atrophy was found in one case, with the largest diameter of FTMH. BCVA improved in two cases and remained the same in two cases. In OCT-A, the area of foveal avascular zone (FAZ) was larger, and foveal vessel density (FVDS) was smaller in eyes after secondary PPV in comparison to fellow eyes. In microperimetry, retinal sensitivity was lower in eyes after secondary PPV, and eccentric fixation was found in 2 of 4 patients.

**Conclusion:**

Although the anatomical results of repeated surgeries of FTMH with ILM plug are favorable, visual function results may be limited. Secondary closure of FTMH with ILM plug may lead to atrophy, changes in the macular vasculature, and eccentric fixation. The trial is registered with NCT03701542.

## 1. Introduction

Pars plana vitrectomy (PPV) is the most effective treatment of full-thickness macular hole (FTMH) [1, 2]. The most untreated FTMHs will progress in size and grade and lead to increasing central visual loss [3]. The classical surgical procedure was first described by Kelly and Wendel in 1991 and consisted of PPV, peeling of epiretinal membranes at the macula, gas tamponade, and face-down positioning for 1 week after surgery [4]. During the next years, as an additional maneuver to macular hole surgery, internal limiting membrane (ILM) peeling was gained and now is routinely used. ILM peeling is a way of making sure no remnant vitreous will exert tangential traction over the macula and provide a support for cell proliferation and the formation of epiretinal membrane [5, 6]. Reported closure rates of macular hole following a primary surgical procedure range from 70% to 100% [3]. Better surgical and functional results are associated with the earlier stage of FTMH, better preoperative visual acuity (VA), shorter duration of symptoms, and younger patient age [7]. There are some cases of persistent macular holes, defined as idiopathic macular holes that underwent vitrectomy but were never observed to close in the postoperative period. There is also the less common condition of reopened or recurrent macular hole, where the hole is observed to close after surgery but subsequently reopens [8]. The failure rate of primary surgery in FTMH is less than 10% [9]. For large macular holes, nonclosure rates as high as 44%, even with adequate ILM peeling, have been reported [10]. The reason of this condition may be a residual epiretinal traction, incomplete removal of posterior hyaloid, insufficient gas tamponade, poor compliance by the patient in keeping prone position, or no obvious cause [9]. Diameter of the FTMH over 500 *μ*m is still at risk for surgery failure and poor vision prognosis [5]. The Manchester study showed that standard MH surgical repair with ILM peeling has very high success rate (94%) up to 650 *μ*m. They suggest that the ILM flap technique for macular holes larger than 650 *μ*m should be reserved [11]. Surgery of persistent and recurrent FTMH remains still a challenge, and there is a number of described surgical techniques, which should be considered after failed primary vitrectomy due to FTMH. Analysis of different flap techniques showed a comparable results, so the technique can be chosen based on the surgeon preference [12]. The results of OCT-A and microperimetry in persistent FTMH closed with secondary PPV have not been published so far.

The aim of this study was to analyse the features of OCT-A and microperimetry in patients who underwent secondary PPV due to persistent FTMH with ILM plug.

## 2. Methods

This is a retrospective consecutive case series performed at the Department of General Ophthalmology in Lublin, Poland, between May 2018 and December 2019. The study was performed in accordance with the Declaration of Helsinki. Ethics Committee at the Medical University of Lublin approved this study (approval no. NCT03701542). All participants provided their written informed consent to the study.

Eyes of four patients were included in the study. In two patients, the fellow eye was the healthy eye, and in two remaining patients, the fellow eye successfully closed FTMH after primary PPV. The mean age of patients was 73 years (range 69–79 years). There were 2 female and 2 male subjects. The mean time period between primary and secondary PPV was 5 months. Inclusion criteria were nonclosed FTMH with the primary PPV. Exclusion criteria were as follows: myopia, lamellar macular holes, AMD, diabetic retinopathy, and glaucoma.

In all patients, best corrected visual acuity (BCVA) was measured pre- and postoperatively using the decimal Snellen chart.

Spectral domain optical coherence tomography (SD-OCT) (Optovue, Inc) was performed also pre- and postoperatively. The diameter of FTMH was measured in micrometers (*µ*m) as the largest distance between margins of the hole. To evaluate the visual function after vitrectomy, we used microperimetry MAIA (CenterVue, Italy). The following parameters in microperimetry were analysed: sensitivity and the average threshold (AT), macular integrity, and fixation stability.

OCT-angiography (Optovue, Inc) was performed postoperatively in both eyes. The OCT-A measurements (6 × 6) were performed after secondary vitrectomy. The following parameters in OCT-A have been analysed: area of superficial foveal avascular zone (FAZ) and the foveal vessel density (FVDS).

### 2.1. Surgical Procedure

At first, patients were operated with the standard surgical procedure consisted of 23-gauge PPV (Constellation, Alcon Surgical, Fort Worth, USA), ILM peeling after brilliant blue dying (Brilliant Peel, Fluoron, Germany) or indocyanine green (ICG), SF_6_ tamponade, and face-down positioning. All eyes were pseudophakic, as cataract surgery was performed before vitrectomy. During secondary PPV, autologous transplantation of the ILM was performed (Video, Supplemental Digital Content 1). All surgeries were performed by the same surgeon (KN). ILM was stained again with brilliant blue or ICG for 60 seconds. A plug of ILM was peeled from the periphery of the posterior pole. The flap of ILM of a diameter approximately 1DD was transferred to the macular hole prior to the air-fluid exchange and subsequently covered the hole. No viscoelastic nor perfluorocarbon liquid was used to stabilize the plug. Patients received 12% perfluoropropane (C_3_F_8_) gas as a tamponade and were postured face down after vitrectomy.

## 3. Results

The mean follow-up period was 6 months (range 3–11 months). ILM flap was integrated in all cases (100%) after secondary PPV with ILM plug, and FTMH was closed with *V*-shape. BCVA was improved after secondary PPV in 2 of 4 cases. Mean visual acuity before secondary PPV was 0.08 (range 0.05–0.1) and postoperatively 0.16 (range 0.05–0.3) (Table 1).

The mean time period from the diagnosis to surgery was 7 months (range 6–8 months). The mean diameter of FTMH was 577 *µ*m before first PPV and 720 *µ*m after first unsuccessful PPV. One case (Case 3) presented macular atrophy observed in OCT after the secondary PPV.

Mean FAZ after secondary PPV was equal to 0.409 mm^2^ and was larger than in the fellow eye (0.282 mm^2^). Eyes after one PPV also presented larger FAZ measurement (0.287 mm^2^) than the healthy eye (0.180 mm^2^). Mean FVDS measured in SCP in eyes operated twice was 14.38% and 23.88% in the fellow eyes.

Overall, functional results of secondary PPV obtained by microperimetry were as follows: mean AT was equal to 18.8 dB, in comparison to 26.1 dB in the fellow eye, fixation was relatively unstable, and eccentric fixation was found in 2 of 4 patients: *P*1 = 74%, *P*2 = 96% and *P*1 = 42%, *P*2 = 85%.

Detailed results obtained by each of four patients of case series are presented below.

### 3.1. Case 1

**Figure 1:**
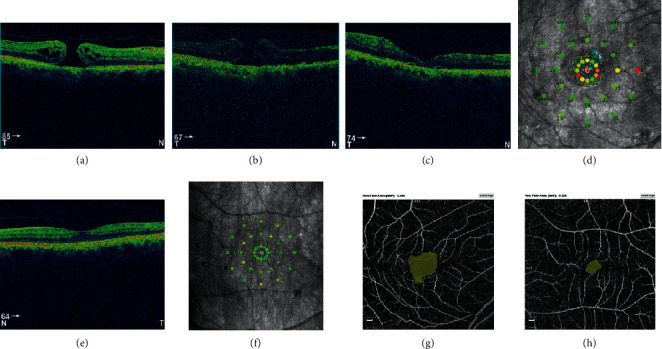
(a) Full-thickness macular hole (FTMH) in the right eye before surgery. Hole diameter equal to 475 *µ*m. (b) Unclosed FTMH in the right eye 1 month after vitrectomy. Poor image quality caused by the rest of SF_6_ gas. Hole diameter equal to 452 *µ*m. (c) Closed FTMH 3 months after secondary vitrectomy in the right eye. (d) Postoperative microperimetric results of the right eye. (e) OCT image of macula of the healthy, left eye. (f) Normal microperimetry result of the left eye. Postoperative FAZ measurement using a nonflow software. Foveal avascular zone (FAZ) of the right eye (g) twice operated, increased significantly compared to FAZ of the healthy, left eye (h).

A male patient, 69 years old, presented in the right eye a FTMH, and the fellow eye was normal. The diameter of FTMH at presentation was 475 *µ*m in OCT measurements. BCVA before surgery was 0.1. Eight months later, he underwent vitrectomy, and one month after surgery, BCVA was 0.1, and fundoscopy revealed unclosed FTMH, which was confirmed in OCT. Patient decided for the secondary surgery. Six months later, PPV was again performed, and autologous transplantation of the ILM was performed. It was a successful surgery, and *V*-shape closure of FTMH was received (Figure 1). We observed improvement in the BCVA 3 months after the surgery to 0.3. In microperimetry, AT was 24.9 dB and classified as suspect (between 23 and 25 dB). Fixation was stable (*P*1 = 92% and *P*2 = 100%). Macular integrity was abnormal (99.4). The area of superficial foveal avascular zone (FAZ) was 0.324 mm^2^, and FVDS measured in superficial capillary plexus (SCP) was 18.3%. In the fellow, the normal eye BCVA was 0.9. OCTA parameters were as follows: FAZ equal to 0.180 mm^2^ and FVDS was 28.8%. Microperimetric results were as follows: AT was 27.0 dB and was classified as normal, and fixation was stable (*P*1 = 96% and *P*2 = 100%).

### 3.2. Case 2

A female patient, 72 years old, with diagnosis of FTMH in the right eye. The preoperative BCVA was 0.1. She was operated 5 months later, and 2 months after successful PPV, BCVA of the right eye was 0.5 and 3 years later was 0.8. Microperimetric parameters were as follows: AT was 27.9 dB and was classified as normal, and fixation stability was relatively unstable (*P*1 = 66% and *P*2 = 89%). Macular integrity was suspicious (49.8). The results of postoperative OCTA images were as follows: the superficial FAZ was 0.158 mm^2^ and FVDS was 33.6%.

**Figure 2:**
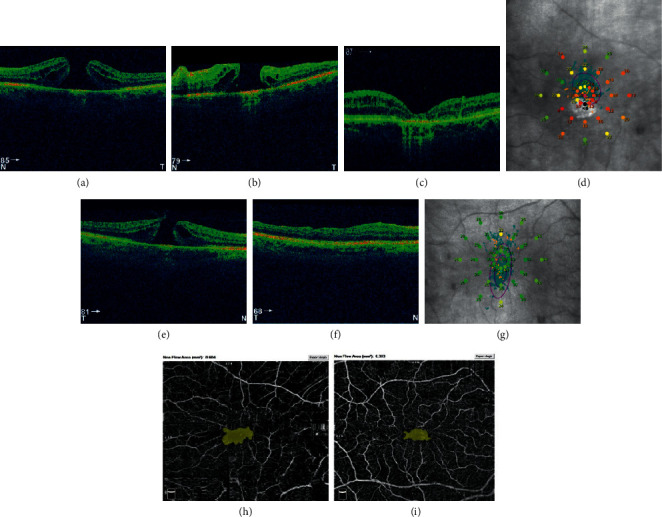
Left eye of the patient no. 2. (a) FTMH before surgery. Diameter equal to 485 *µ*m. (b) Unclosed FTMH after vitrectomy with diameter 765 *µ*m. (c) Closed FTMH with intraretinal cysts, 6 months after secondary PPV. (d) Postoperative microperimetric results. FTMH of the right eye of patient no. 2 before (e) and after primary, successful pars plana vitrectomy (PPV) (f). (g) Postoperative microperimetric results of the right eye. (h) Postoperative FAZ of left eye, operated twice. Nonflow area: 0.604 mm^2^, BCVA 0.1. (i) Postoperative foveal avascular zone (FAZ) of the right eye after one vitrectomy, nonflow area: 0.363 mm^2^, BCVA equal to 0.8.

Two years later, the FTMH in the left eye was diagnosed. The preoperative BCVA of the left eye was 0.1 and diameter was 485 *µ*m. The left eye was operated after 8 months from diagnosis. One month after primary vitrectomy, there was no BCVA improvement, and OCT image showed unclosed FTMH with increased diameter equal 765 *µ*m. Two months later, the secondary vitrectomy was performed with anatomical success, and *V*-shape closure was observed (Figure 2). After 3 months, the BCVA was 0.1. Microperimetric parameters were as follows: AT was 17.7 dB and was classified as suspect, fixation was relatively unstable (*P*1 = 74% and *P*2 = 96%), and eccentric fixation was found. The results of postoperative OCTA images present that the superficial FAZ was 0.316 mm^2^ and FVDS was 21.9%.

### 3.3. Case 3

**Figure 3:**
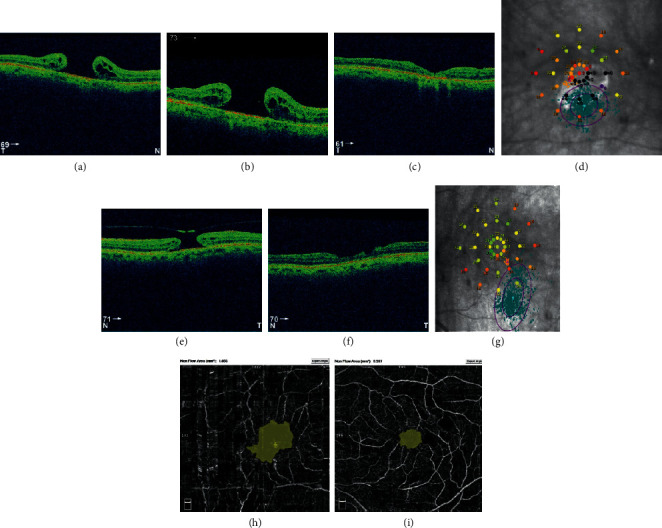
(a) Preoperative FTMH of the right eye of patient no. 3 with diameter 765 *µ*m. (b) Unclosed FTMH after primary vitrectomy, 940 *µ*m of diameter. (c) After secondary vitrectomy with atrophy. (d) Postoperative microperimetric results, BCVA 0.05. FTMH of the left eye with diameter 660 *µ*m before (e) and after primary, successful vitrectomy (f). (g) Postoperative microperimetric results, BCVA 0.1. (h) Postoperative foveal avascular zone (FAZ) of the right eye, nonflow area: 1.856 mm. (i) Postoperative FAZ of the left eye, nonflow area: 0.557 mm^2^.

A female patient, 79 years old, with FTMH in both eyes. The left eye was operated at first, 5 months after clinical diagnosis, with anatomical success. Preoperative BCVA of the left eye was 0.1. Postoperative BCVA of this eye was still 0.1. The functional results of the left eye were as follows: AT was 21.7, fixation was relatively unstable (*P*1 = 32% and *P*2 = 70%), and eccentric fixation was found. Postoperative OCTA images showed that the superficial FAZ was 0.416 mm^2^ and FVDS was 18.5%. 7 months later, the right eye was selected for surgery due to FTMH. Preoperative BCVA of the right eye was 0.05 and the diameter of FTMH in OCT was 765 *µ*m. The primary vitrectomy was unsuccessful, there was no BCVA improvement, and FTMH was still observed in fundoscopy and OCT scans after surgery. Hole diameter postoperatively was 940 *µ*m. Seven months later, secondary vitrectomy was performed and ended with *V*-shape closure of FTMH (Figure 3). 11 months observation of the right eye showed the macular atrophy and following functional results: BCVA was 0.05, AT in microperimetry was 12.8, fixation was relatively unstable (*P*1 = 42%, *P*2 = 85%), and eccentric fixation was found. OCTA images showed after surgery that the superficial FAZ was 0.461 mm^2^ and FVDS was 7.3%.

### 3.4. Case 4

**Figure 4:**
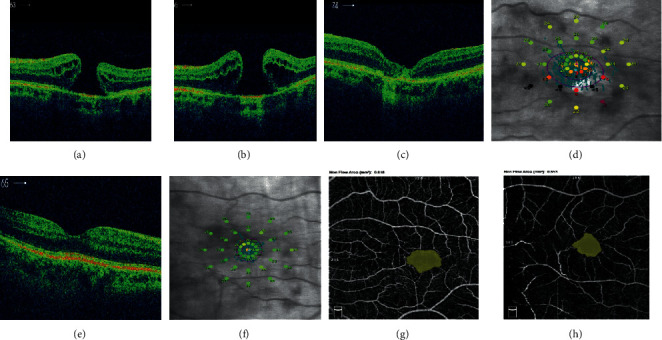
(a) Preoperative FTMH of the left eye of patient no. 4 with diameter 588 *µ*m. (b) Unclosed FTMH 1 month after vitrectomy, hole diameter equal to 726 *µ*m. (c) *V*-shaped closure of FTMH after secondary vitrectomy. (d) Postoperative microperimetric results of the left eye. (e) OCT image of macula of the healthy, right eye. (f) Normal microperimetry result of the right eye. (g) Postoperative FAZ of the left eye, nonflow area: 0.818 mm^2^. (h) FAZ of the right eye, nonflow area: 0.653 mm^2^.

A male patient, 72 years old, presented a FTMH in the left eye, and the fellow eye was normal. The diameter of FTMH at presentation was 588 *µ*m in OCT measurements. BCVA before surgery was 0.1. He was operated 6 months after making diagnosis. After vitrectomy with ILM peeling, BCVA was still 0.1. Unclosed FTMH was observed in fundoscopy, and in OCT examination, the hole diameter was 726 *µ*m. Patient decided for the secondary surgery. Four months after primary PPV, the secondary surgery was again performed, and autologous transplantation of the ILM was performed. *V*-shape closure of FTMH was received, and BCVA was improved to 0.2 (Figure 4). Microperimetry parameters were as follows: AT was 20.0 dB and classified as suspect (between 23 and 25 dB). Fixation was relatively unstable (*P*1 = 47% and *P*2 = 89%). Macular integrity was abnormal (99.4). The area of the superficial foveal avascular zone (FAZ) was 0.537 mm^2^, and FVDS measured in superficial capillary plexus (SCP) was 9.8%. In the fellow, healthy eye BCVA was 0.9. OCT-A parameters were as follows: FAZ equal to 0.374 mm^2^ and FVDS 14.6%. Microperimetric results were as follows: AT was 27.9 dB and was classified as normal, fixation was stable (*P*1 = 96% and *P*2 = 97%), and macular integrity was normal (27.9).

## 4. Discussion

PPV combined with ILM peeling has been a gold surgical standard in the treatment of FTMH for many years. Closure rate is significantly higher (90%) after ILM peeling compared with the non-ILM peeling technique (58%) [5]. It is considered that ILM peeling provides good anatomic and functional results in FTMH by completely releasing the traction forces on the macula and reducing the postoperative epiretinal membrane formation [5]. Four main types of possible macular hole closure after PPV with ILM peeling have been described, which correlate with postoperative outcome. They are distinguished as follows: *U*-shape closure with the normal foveal contour and central foveal depression, which is associated with the best functional results, *V*-shape closure, which demonstrates the steep foveal contour, and *W*-type closure with irregular foveal defect and flat-closed macular holes [13].

Unfortunately, unclosure of the FTMH after primary vitrectomy still can occur, even performed by experienced surgeons. The reasons for no successful primary vitrectomy may be diverse. The diameter of FTMH over 500 *μ*m is considered a risk factor for unclosure after primary vitrectomy [5]. In our case series, the mean diameter of FTMH before primary vitrectomy was 577 µm; thus, it seems to be the main reason for unclosure of the FTMH.

Moreover, Wendel et al. suggest better improvement in visual acuity of patients undergoing FTMH repair with less than 6 months' duration of visual symptoms [14]. In our patients, the mean time between clinical diagnosis and first unsuccessful vitrectomy was 7 months, so we can suspect that delay from preoperative assessment to surgery may be also the reason of failure of the first vitrectomy due to FTMH.

Repeated surgeries for unclosed FTMH have a lower success rate than primary surgery [5]. However, in our case series, the secondary PPV with autologous ILM plug was an effective surgical procedure to receive the closure of persistent FTMH. We achieved the closure of FTMH after secondary surgery in all four cases (100%), although it is a small case series. Two of the described above patients presented bilateral FTMH, one eye was operated with anatomical success, and the second one failed to close after primary surgery, thus secondary PPV was performed. Reported secondary closure rate of revision surgeries was equal 76% [8]. Yek et al. and colleagues observed 85% closure rate of FTMH after secondary vitrectomy [8].

Modern approach for closing persistent FTMH is to use a technique called autologous transplantation of the ILM, introduced by Morizane et al. [15]. A plug of ILM is peeled from the periphery of the posterior pole with a size big enough to insert into the macular hole. It can be applied to eyes with FTMH, in which the initial vitrectomies with ILM peeling fail to achieve closure, or to eyes with secondary macular holes, in which the ILM already has been removed in previous vitrectomies [15]. The flap of ILM is transferred to the macular hole prior to the air-fluid exchange. A challenge of this technique is the handling of the free ILM patch and the risk of dislocation of ILM patch during air-fluid exchange [16]. The other possible intraoperative complication is the damage to the RPE at the base of the macular [5, 17]. Free ILM flap transplantation was also proved by Ma and coauthors in their study to be effective to achieve anatomical and functional improvement for primary treatment of large macular hole [5]. They advise to respect the original orientation of the free ILM flap because of retinal surface, which is rougher than the vitreous surface and potentially providing stronger hold to RPE layer [5]. Chang and colleagues recommend the other surgical technique, first proposed by Grewal, a neurosensory retinal free flap transplantation, as an alternative surgical technique for the repair of large refractory MHs after unsuccessful ILM peeling surgeries [18]. They presented the technique consisted of a neurosensory retinal free flap with a 1.5–2 MH diameter using whole blood or Viscoat and silicone oil to tamponade the vitreous cavity. The Viscoat or blood was used to help fixing the retinal free flap and additionally contain multiple growth factors [18]. Yek et al. achieved a high secondary closure rate of FTMH and good visual outcomes mostly performing PPV with perfluoropropane gas as tamponade, ILM peeling without any additional procedures to close the hole (e.g., inverted flap technique), and face-down positioning [8]. They reported the progressive improvement of VA after secondary vitrectomy, similar to the trend after successful primary surgery [8, 19, 20]. Probably, it is the result of remodeling and regeneration of foveal retina and photoreceptors [8, 21]. Yek et al. demonstrated also a significant increase in diameter of macular hole after failed primary vitrectomy [8]. Our patients also presented larger diameter before revision surgery compared to the size before primary vitrectomy. Some authors to achieve the closure of FTMH have tried alternative endotamponades such as semifluorinated alkane F_6_H_8_, standard silicone oils, or heavy silicone oils [8]. In recent years, a second operation with transforming growth factor beta 2 application or autologous platelet concentrate inserted inside the macular hole was proposed to obtain the closure of FTMH [8].

ILM peeling performed during vitrectomy can lead to focal intraretinal hemorrhages or retinal edema that might affect retinal vasculature [22]. Investigators hypothesised that changes in the structural integrity of macular capillary plexuses might be associated with the postoperative retinal structural and functional changes in eyes with FTMH [23]. Michalska-Małecka et al. described in their article the OCTA features of FTMH before and after surgical treatment. In their study, OCTA showed enlargement of the superficial foveal avascular zone (FAZ) and increased central retinal thickness (CRT) in foveal area before the surgery. Mean preoperative FAZ area was 0.39 ± 0.07 mm^2^, and mean CRT was 396 ± 62.6 *µ*m. After vitrectomy, both parameters were reduced; mean FAZ area was 0.24 ± 0.07 mm^2^, and mean CRT was 272 ± 30.7 µm. They observed also postoperatively a statistically important increase of fovea vessel density (FVDS) in superficial capillary plexus (SCP). Mean FVDS in SCP was 29.84% ± 4.17% preoperatively and increased to 35.02% ± 3.47% postoperatively. They found a relatively strong correlation between preoperative FAZ and postoperative BCVA [24]. In our patients, the best results in OCTA images presented Case 2 with the lowest FAZ and the highest FVDS. The worst postoperative results we observed in Case 3 with the high FAZ and the lowest FVDS presented as wide ischemic areas in vascular density color perfusion maps. Case 4 presented the highest value of FAZ and low FVDS, although we observed better functional result, BCVA and stable fixation. Some authors have described retinal fibrosis and pigment epithelium dystrophy in the macular area, after internal limiting membrane autologous transplantation, which can affect final visual recovery [8]. We observed postoperative atrophic changes in Case 3. Our patients presented higher value of FAZ in eyes operated twice than to the eyes operated once. Cho et al. compared OCTA features of retinal microvasculature in eyes with surgically closed FTMH with their fellow eyes. They reported that, based on the postoperative OCTA images, the mean FAZ area in both the SCP and deep capillary plexus (DCP) was significantly smaller than those for the fellow eyes. To close the FTMH, tissues are brought centrally, and the FAZ may become smaller [22]. They also found that the vascular density (VD) ratio in the DCP was lower than that for the fellow eyes, suggesting a possible vulnerability to tractional stress in the DCP [11]. Kim et al. concluded the same in their study [23]. In consideration, it should be noted that FAZ area increases on average 1.48% per year and FTMH mostly affects elderly people [25]. Sul et al. analysed the choroidal thickness (CT) in macular holes to determine the association between CT and anatomic success after surgery. The choroid plays the main role in blood supply, metabolic activity of fovea, and changes in the blood flow, which may modify the healing process. They found that CT was significantly lower in eyes with MH than fellow eyes and preoperative subfoveal CT difference between open and closed MHs after vitrectomy. Preoperative subfoveal CT was thinner in open MHs, but there was no association with anatomic success. Authors concluded that choroidal thinning is likely the result of chronic and larger MHs, which have worse prognosis of closure after vitrectomy [26].

Previous studies have reported characteristics of OCTA images in eyes with FTMH or comparison of OCTA images before and after surgery [22–24]. Analysis of OCTA images in eyes after secondary vitrectomy due to FTMH should be performed in a bigger sample of patients and compared with the group of patients with successful first surgery.

The secondary closure of FTMH may be associated with the prolonged proliferation of glial tissues in the fovea with fibrotic and depigmentation phenomena [27].

To evaluate functional outcomes of FTMH surgery, microperimetry is very useful as rapid, safe, noninvasive examination [21, 28]. Tarita Nistor et al. suggest that the closure of FTMH could lead to a complex reorganization of fixation behaviour [29]. Sborgia et al. in their article proposed that parameters such as preoperative macular sensitivity (MS) and bivariate contour ellipse area (BCEA) have a predictive role on postsurgical VA [30]. BCEA is more accurate estimation of the fixation pattern, which is calculated as an ellipse that covers fixation eye positions and takes into account 1 or 2 times the standard deviation, including consequently 95% (BCEA95) and 63% (BCEA63) of points [18]. In FTMH, a lower sensitivity at the central 4° of the macula (CMS) can be observed as an absolute scotoma and caused by the neurosensory defect. The higher sensitivity at 12° (MS) is observed in the region of the retina around the hole. Analysis of changes in MS showed that both CMS and MS significantly improved after surgery. Authors suggest that retinal sensitivity at 12° (MS) is less influenced by foveal microstructure recovery after macular hole closure than CMS at central 4° [29]. Results shown by Bonnabel et al. prove the correlations between MS and postoperative VA. They also concluded that postoperative outer retinal layer integrity is associated with better final retinal sensitivity [31]. In our patients, the best functional result was presented in Case 1, with improvement of BCVA and stable fixation, probably because of the small diameter of FTMH. Postoperatively, the visual acuity and microperimetric results were the poorest in Case 3, which presented preoperatively the largest diameter of FTMH. BCVA was 0.04, and we observed the absolute scotomas, which correspond to the atrophy of neurosensory retina. This is probably a reason of eccentric fixation. Eccentric fixation was also found postoperatively in Case 2, despite the better BCVA than Case 3. Unfortunately, we do not have preoperative microperimetric data to compare the pattern of fixation, as microperimetry was not performed in our case study before vitrectomy due to FTMH. It is difficult to assess if the eccentric fixation is a result of surgical trauma or was presented before the vitrectomy as a result of FTMH. In the fellow eyes operated once, the fixation was unstable, but there was no eccentric fixation. It is already known that ILM peeling in normal retina will not decrease the retinal function in a short-term after surgery, and we suppose that eccentric fixation is the effect of closure of the hole with ILM plug during secondary vitrectomy [32]. Sometimes, it needs many attempts to put the ILM plug directly to the macular hole during the secondary vitrectomy; thus, we suppose that the contact of the instrument with the retina may lead to atrophy and poorer visual outcomes.

The usability of microperimetry is proved in many articles, and authors put effort to find potential predictive factors for assessment of future and existing treatment [33, 34], so it seems reasonable to perform it before vitrectomy. In a recent study of Kunikata and colleagues, microperimetry was performed preoperatively and 1, 3, and 6 months postoperatively in 21 patients with primary FTMH. Lower recovery of retinal sensitivity was found in the superior sector of the macula [35]. It was also already shown that the preoperative retinal sensitivity is related to the diameter of the FTMH and retinal architecture (the width of the perifoveal cystic cavities and the area of choroidal transparency) [36].

In the longer follow-up period, we could observe how macular sensitivity and fixation stability change and assess the surgical efficacy and find the best surgical technique; thus, further prospective long-lasting studies with preoperative assessment are needed to assess the visual function after secondary vitrectomy with ILM plug.

The limitation of our study is small number of patients included, but they were collected during the long period, as the rate of unclosed FTMH is quite small, and these are rare cases.

In conclusion, the secondary PPV with autologous ILM plug was an effective surgical procedure to receive the closure of persistent FTMH, although visual function results may be limited. Characteristics of OCTA images in eyes with FTMH or comparison of OCTA images before and after PPV have not been done so far, but they should be performed in a bigger sample of cases.

## Figures and Tables

**Table 1:** The results of time period before primary PPV, visual acuity (BCVA) in both eyes, diameter of the hole in OCT before and after primary vitrectomy in eyes operated twice (EOT), microperimetry parameters (average threshold (AT) and fixation pattern) and OCT-A parameters (foveal avascular zone (FAZ), and foveal vessel density (FVDS) in EOT with pars plana vitrectomy (PPV) due to persistent full-thickness macular hole (FTMH) and fellow eyes (FE).

	Time period from diagnosis to surgery (months)	BCVA		Diameter of the hole (*µ*m)		AT (dB)		Fixation pattern		SD-OCT	FAZ (mm^2^)		FVDS (%)	
		EOT	FE	Before first PPV	After first PPV	EOT	FE	EOT	FE	EOT	EOT	FE	EOT	FE
Case 1	8	0.3	0.9 healthy	475	452	24.9	27.0	Stable	Stable	*V*-shape	0.324	0.180	18.3	28.8
Case 2	8	0.1	0.8 operated once	485	765	17.7	27.9	Unstable, eccentric fixation	Unstable	*V*-shape	0.316	0.158	21.9	33.6
Case 3	7	0.05	0.1 operated once	760	940	12.7	21.7	Unstable, eccentric fixation	Unstable	*V*-shape, atrophy	0.461	0.416	7.5	18.5
Case 4	6	0.2	0.9 healthy	588	726	20.0	27.9	Unstable	Stable	*V*-shape	0.537	0.374	9.8	14.6

## Data Availability

The data used to support the findings of this study are available from the corresponding author upon request.

## Supplementary Materials

Supplementary Materials Video showing surgery.
